# Drawbacks of Dialysis Procedures for Removal of EDTA

**DOI:** 10.1371/journal.pone.0169843

**Published:** 2017-01-18

**Authors:** Andreia Mónico, Eva Martínez-Senra, F. Javier Cañada, Silvia Zorrilla, Dolores Pérez-Sala

**Affiliations:** 1 Department of Chemical and Physical Biology, Centro de Investigaciones Biológicas, C.S.I.C., Madrid, Spain; 2 Department of Cellular and Molecular Biology, Centro de Investigaciones Biológicas, C.S.I.C., Madrid, Spain; Russian Academy of Medical Sciences, RUSSIAN FEDERATION

## Abstract

Ethylenediaminetetraacetic acid (EDTA) is a chelating agent commonly used in protein purification, both to eliminate contaminating divalent cations and to inhibit protease activity. For a number of subsequent applications EDTA needs to be exhaustively removed. Most purification methods rely in extensive dialysis and/or gel filtration in order to exchange or remove protein buffer components, including metal chelators. We report here that dialysis protocols, even as extensive as those typically employed for protein refolding, may not effectively remove EDTA, which is reduced only by approximately two-fold and it also persists after spin-column gel filtration, as determined by NMR and by colorimetric methods. Remarkably, the most efficient removal was achieved by ultrafiltration, after which EDTA became virtually undetectable. These results highlight a potentially widespread source of experimental variability affecting free divalent cation concentrations in protein applications.

## Introduction

Metal chelators, such as EDTA, are widely used for inhibition of proteases during protein purification or during preparation of cell or tissue extracts [1]. EDTA is also employed in procedures to eliminate endotoxin from certain protein preparations [2] or to prevent oxidation by metals [3]. However, the presence of EDTA in biological samples, even at low concentrations, may interfere with assays like those aimed at assessing the effects of divalent cations on protein or cellular functions and hence, it is often necessary to remove the chelator. Dialysis or gel filtration are routinely used in research articles [2, 4] and recommended in commercial technical application notes for exchange or removal of low molecular weight buffer components, including EDTA, from cell or tissue extracts or protein preparations. Dialysis is also preferred when the volume of protein available or its concentration are too low for size exclusion chromatography with columns long enough to render a good separation. Moreover, dialysis is usually the method of choice when the protein is purified from bacterial inclusion bodies and the denatured protein needs to be refolded prior to the experiments by this procedure [5, 6]. This is the case of intermediate filament proteins, which are typically purified using buffers containing EDTA. In addition, they require solubilization either from inclusion bodies, in the case of recombinant proteins expressed in bacteria, or from insoluble eukaryotic cell cytoskeletal fractions, for which high urea concentrations, up to 9.5 M, are used [7–9]. Later, the denatured proteins are refolded by step-wise dialysis to gradually remove urea, usually in low salt buffer to keep the protein unassembled, as high ionic strength triggers polymerization [8]. In fact, intermediate filament proteins such as desmin, vimentin or glial fibrillary acidic protein (GFAP) purified through these methods, have been widely studied to assess polymerization or association changes induced by increasing ionic strength or by divalent cations, usually at millimolar concentrations [9, 10].

Nevertheless, confirmation of adequate elimination of additives used during purification or refolding is necessary for subsequent applications, above all, when studying the impact of low concentrations of various ligands on protein structure or function. Here we show that EDTA can be carried on unnoticed during protein dialysis. Moreover, we provide simple methods for its detection and improved removal.

## Materials and Methods

### Materials

Amicon ultrafiltration devices (10K cut off) were from Millipore. PD-SpinTrap G-25 columns were from GE Healthcare. Slide-A-Lyzer MINI Dialysis devices (20K cut off) were from Thermo. 96-well plates were from Falcon. Recombinant hamster vimentin was from Cytoskeleton, Inc. Recombinant human vimentin was from Biomedal (Sevilla, Spain). Other reagents were of the highest quality from Sigma.

### Protein dialysis

Bovine serum albumin (BSA) at 1 mg/ml in 5 mM Pipes, pH 7.0 containing 1 mM EDTA was dialyzed against 5 mM Pipes, 1 mM DTT, pH 7.0 with four buffer changes and, subsequently, into 5 mM Pipes, 0.25 mM DTT, pH 7.0 with two changes. Dialysis of vimentin followed the standard refolding protocol [8]. Briefly, the human recombinant protein at 1 mg/ml in 5 mM Tris-HCl, pH 7.6, containing 8 M urea, 1 mM EDTA, 10 mM β-mercaptoethanol, 0.4 mM PMSF and approximately 150 mM KCl, was subjected to step-wise dialysis against 5 mM Pipes pH 7.0, 1 mM DTT containing 6 M urea, then 4 M urea, 2 M urea and no urea at r.t., and finally, to two additional steps against 5 mM Pipes, pH 7.0, 0.25 mM DTT, the last one for 16 h at 16°C.

### Spin-column gel filtration and ultrafiltration

Gel filtration was conducted using PD SpinTrap G-25 1 ml columns equilibrated with 5 mM Pipes, 0.1 mM DTT, pH 7.0, before loading 140 μl samples of vimentin or BSA and eluting according to the instructions of the manufacturer. For ultrafiltration, BSA or vimentin samples (250 μl) containing 1 mM EDTA were diluted 10-fold with EDTA-free buffer, applied to Millipore Amicon Ultra filter units (10 K pore size) and centrifuged at 3000xg for 15 min at 16°C, which concentrated the samples down to their original volume. Then, samples were diluted again 10-fold with buffer without EDTA and the procedure was repeated.

### Colorimetric estimation of EDTA concentration

The concentration of EDTA in the protein solutions was estimated through a colorimetric assay by monitoring its competition with 4-(2-pyridylazo)-resorcinol (PAR) for Zn binding. The binding of zinc to PAR forms a colored complex with absorbance at 492 nm [11]. The presence of EDTA induces a decrease in the formation of this complex. A calibration curve was obtained by titration of samples containing 100 μM PAR and 10 μM Zn with known concentrations of EDTA and measuring the absorbance at 492 nm, using a Varioskan Flash (Thermo) microplate reader. The amount of EDTA in the protein preparations was determined from their absorbance at 492 nm after incubation with 100 μM PAR and 10 μM Zn, and extrapolation using the calibration curve. Sample volumes of 1 to 10 μl were used in a typical total assay volume of 100 μl. Measurements were performed 5 min after mixing of reagents.

### NMR analysis

NMR spectra were acquired in a 500 MHz Bruker AVANCE equipped with a SEF 19F-1H probe or a 600MHz Bruker AVANCE equipped with a cryogenic triple resonance TXI probe. In the case of samples dissolved in deuterated buffer (20 mM deuterated Tris, Cambridge Isotope, UK), spectra were acquired with a simple 90° pulse sequence (zg Bruker pulse sequence) and with 32K data points and 2 second recovery delay and 10 ppm of spectral width centered at 4.7 ppm (chemical shift of residual HDO signal). In the case of samples dissolved in non-deuterated buffers, a 10% volume of deuterated water was added for locking deuterium signal and the standard Bruker pulse sequence “zgesgp” using excitation sculpting gradients for water signal suppression was used. The spectra were acquired at 25°C with 32K points, 2 second recovery delay and 14 ppm of spectral width centered at 4.7 ppm (chemical shift of water). The spectra were acquired using from 8 to 2048 scans depending on the sample concentration; no line broadening was applied in the processing. Bruker TOPSPIN software was used for acquisition and processing the spectra. For testing the pH dependency of chemical shift signals of EDTA, samples of 1 to 3 mM EDTA, with and without cation (Ca^2+^, Zn^2+^, Mg^2+^ or La^3+^), in 20 mM deuterated Tris were prepared at different pH (between 5 and 9) by acidification with deuterated hydrochloric acid. The actual pH was measured after the addition of the corresponding dichloride salt of each cation. EDTA and ZnCl_2_ solutions were prepared in deuterated water.

## Results and Discussion

**Fig 1:**
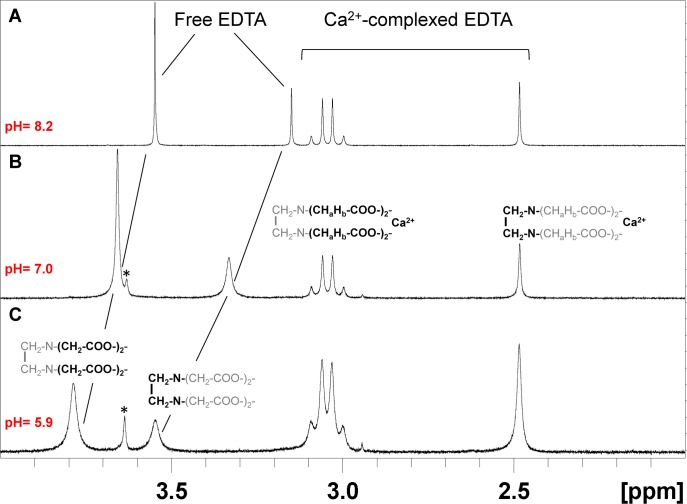
Proton NMR spectra of EDTA and its Ca^2+^-complexed forms at different pH. EDTA (3 mM) and CaCl_2_ (1.5 mM) were mixed in deuterated Tris buffer to yield equimolecular amounts of free and Ca^2+^-complexed EDTA. Proton NMR spectra of the mixture were acquired at different pH: (A) pH 8.2, (B) pH 7.0, (C) pH 5.9. The signals corresponding to protons of the free and Ca^2+^-complexed forms of EDTA are indicated. The NMR spectrum of EDTA alone is identical to that of free EDTA presented. The moieties responsible for each of the components of the signals are depicted in bold. The signal corresponding to Tris buffer is labeled with *.

We have recently explored protein modification by electrophilic lipids using both cultured cells and commercially available protein preparations, including the IF protein vimentin [12, 13]. In order to study the effect of micromolar concentrations of divalent cations on these processes we set out to rule out the presence of metal chelators in the protein preparation. For this we first conducted NMR experiments. Proton NMR has been previously used for detecting either EDTA or divalent cations as their EDTA chelates in blood samples obtained with EDTA as anticoagulant [14, 15]. In addition, NMR methods have been employed to quantitate metals in human serum upon addition of exogenous EDTA [16]. As shown in Fig 1, free EDTA gives two singlet peaks, the one at low field corresponding to the 8 methylene protons of acetyl moieties, and the one at high field to the 4 protons of the ethylenediamine moiety. The shifts of these peaks are strongly dependent on the pH of the sample and their signals show a significant broadening at low pH (Fig 1). Moreover, complexation of EDTA with different metals gives rise to characteristic patterns, where the methylene protons of acetyl moieties become un-equivalent, resulting in differentiated signals due to the characteristic structures of the chelates, as depicted in Fig 1 for the EDTA-calcium complex. This ensures the specificity of the detection and minimizes pH dependence, since the shifts of the signals of the complexed EDTA form appear constant in this pH range (Fig 1 and [16]).

**Fig 2:**
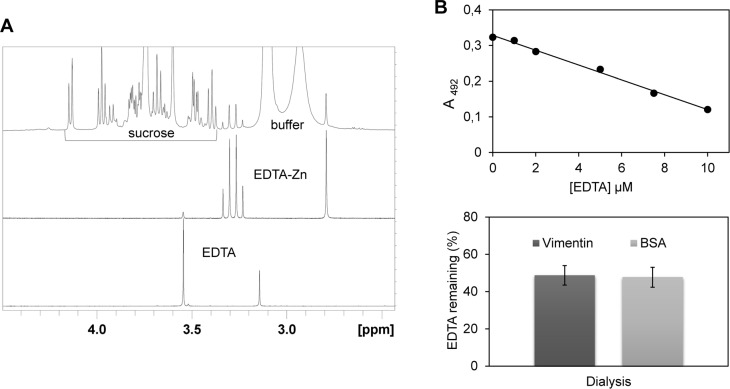
Detection and quantitation of EDTA in protein samples. (A) NMR analysis. Upper panel: 500MHz 1D-proton NMR spectrum of a commercial sample of vimentin (0.7 μM) with added 150 μM ZnCl_2_. Signals for the protons of sucrose, a stabilizer present in the sample as indicated in specifications, and those of PIPES buffer are easily identified. An additional singlet at 2.79 ppm and two coupled doublets at 3.25 and 3.32 ppm (2JHH = 17.3 Hz) are also observed. Middle panel: reference proton spectrum of EDTA in presence of Zn^2+^, the two coupled doublets corresponding to the AB spin system of the methylene protons of the four acetyl groups, non-equivalent due to the structure of the metal chelate, and the singlet corresponding to the four equivalent protons of the ethylenediamine moiety are apparent. Lower panel: reference proton spectrum of EDTA at pH 7.2, only the two characteristic singlets of uncomplexed EDTA appear. (B) Detection of EDTA by colorimetric analysis. Upper panel: Calibration curve showing the dependence on EDTA concentration of the absorbance at 492 nm of mixtures containing 100 μM PAR and 10 μM ZnCl_2_. Lower panel: Amount of EDTA remaining in samples from vimentin and BSA subjected to extensive dialysis as determined from the absorbance at 492 nm after incubation with PAR and ZnCl_2_, using the calibration curve. Initial EDTA concentration in the samples was 1 mM. Data shown are mean ± SD of 4 assays.

Therefore, protein samples were analyzed after adding known amounts of a divalent cation such as calcium or zinc, the latter case being illustrated in Fig 2. This revealed the presence of a substantial amount of EDTA in a commercial sample, which is presented as a lyophilized preparation that after reconstitution in water should yield a solution of folded protein in 5 mM Pipes pH 7.0, 1 mM DTT, 5% (w/v) sucrose and 1% (w/v) dextran. In order to obtain a preparation more suitable for our studies we subjected a human vimentin sample in buffer containing 8 M urea, 5 mM Tris-HCl pH 7.6, 1 mM EDTA, 10 mM β‑mercaptoethanol, 0.4 mM PMSF and approximately 150 mM KCl to the typical renaturation dialysis procedure, which included four sequential steps with at least six changes against EDTA-free buffers [8]. NMR analysis of the protein solutions thus obtained showed the retention of EDTA after dialysis (Fig 2A).

A complementary colorimetric assay was also used to further confirm the presence of EDTA and to estimate its levels in the protein preparations after dialysis. For this we took advantage of the ability of the compound 4-(2-pyridylazo)-resorcinol (PAR) to form a colored 2:1 complex with zinc with high affinity (effective dissociation constant of the Zn(PAR)_2_ complex, at pH 7.0, 2.1 x 10^−12^ M^2^ [17]), which presents an absorption maximum at 492 nm and can be measured spectrophotometrically. In turn, EDTA forms a 1:1 complex with zinc of extremely high affinity: 6 x 10^−14^ M at pH 7.0 [18]. Therefore, solutions containing EDTA should compete with PAR for zinc binding, in such a way that the decrease in the absorption at 492 nm measured at the equilibrium can be used to infer the concentration of EDTA present in the sample. For this, we titrated known amounts of EDTA into 100 μM PAR and 10 μM zinc assay mixtures and measured the absorbance at 492 nm to build a calibration curve (Fig 2B, upper panel). We then assayed the EDTA present in the dialysates from their absorbance when mixed with the above specified concentrations of PAR and zinc. These measurements evidenced that samples of vimentin or bovine serum albumin (BSA) originally containing 1 mM EDTA still retained ~450 μM EDTA after dialysis, in good agreement with parallel determinations by NMR (Fig 2A). Therefore, EDTA removal can be easily and conveniently monitored by this procedure. Nevertheless, it should be taken into account that other metal-binding compounds or proteins present in the sample may also compete with PAR for zinc binding, for which detection of EDTA by several methods is advisable. EDTA was also not removed from solutions of other intermediate filament proteins such as GFAP or desmin treated under similar conditions (unpublished observations).

**Fig 3:**
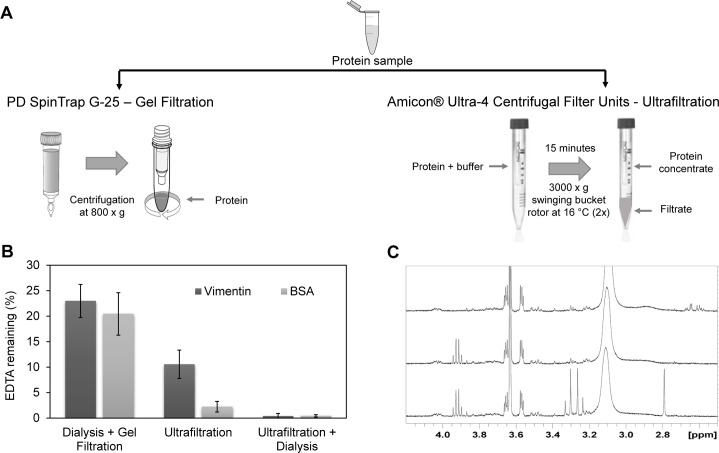
Procedures for EDTA removal from protein samples and detection of EDTA. (A) The protein samples containing 1 mM EDTA were subjected to the indicated purification procedures. Left panel: Samples of BSA or vimentin were subjected to spin column gel filtration as detailed in methods. Right panel: protein samples were applied to Millipore Amicon Ultra filter units (10 K pore size) and subjected to two rounds of ultrafiltration, as described in the text. (B) Colorimetric determination of EDTA present in protein samples after diverse purification procedures using the PAR competition assay. Results shown are mean ± SD of 4 (dialysis plus gel filtration), 2 to 7 (ultrafiltration), or 3 to 7 (ultrafiltration plus dialysis) assays. (C) NMR analysis. Upper panel: 600MHz 1D-proton NMR spectrum of an ultrafiltrated sample of vimentin (1.8 μM final concentration). Signals of buffer and additives used in the purification (glycerol, from the ultrafiltration filters, and DTT) are observed. Protein signals appear at baseline noise level and are not recognizable. Middle panel: The same sample analyzed in the upper panel monitored after addition of 150 μM of ZnCl_2_. A quadruplet that appears at 3.92 ppm corresponds to trifluoroethanol added for referencing. Lower panel: monitorization of the sample after addition of 20 μM EDTA. The signal pattern of the AB system at 3.32 and 3.25 ppm and the singlet at 2.79 ppm corresponding to the Zn^2+^-EDTA chelate are clearly visible.

As the amount of EDTA remaining in the samples was incompatible with many protein studies, we attempted to setup a procedure to effectively remove this chelator. Bearing in mind that the quantity of protein available for biological assays is often limited, we used protocols for small sample volumes, namely, spin column gel filtration and ultrafiltration (Fig 3A). We started by processing the protein solutions already subjected to dialysis by spin-column gel filtration. Although EDTA was further reduced by this treatment, the protein solutions still contained ~200 μM EDTA, as determined by the PAR competition assay (Fig 3B). As an alternative, we turned to ultrafiltration to remove the EDTA. BSA or vimentin samples containing 1 mM EDTA were diluted 10-fold with EDTA-free buffer and ultrafiltrated as indicated in Fig 3A, which concentrated the samples down to their original volume. Then, samples were diluted again 10-fold with buffer without EDTA and the procedure was repeated. Finally, assessment of EDTA content by the colorimetric assay confirmed that EDTA was more effectively removed by this procedure (Fig 3B). At this point, EDTA was not detectable by NMR, limiting its possible concentration to 10 μM or lower in the undiluted protein sample (Fig 3C). A further advantage of this method is that protein dilution can be minimized by monitoring the final retentate volume. Lastly, combination of ultrafiltration and dialysis led to the reduction of EDTA in the protein samples below 5 μM according to the colorimetric assay (Fig 3B).

We have found numerous examples in the literature and in technical notes and protocols where dialysis is employed to remove EDTA. In contrast, evidences on the limitations of this method to completely remove the chelator are scarce and not readily found in bibliographic searches. Our observations together with these previous works [19, 20] indicate that it should not be assumed that EDTA is thoroughly removed by dialysis procedures. Thus, complete removal of EDTA should be assessed either by the procedures described herein or by other available methods, like HPLC or spectrometric procedures [21, 22], as in [19]. The efficiency of EDTA removal by dialysis may vary with buffer composition. In particular, Kuzmenko et al., showed that EDTA removal was poorer when dialysing against buffers with low ionic strength [19], with incomplete removal even after six buffer changes over three days dialysis. To test the influence of ionic strength on the effectiveness of dialysis under our experimental conditions, we have assessed the removal of EDTA (1 mM initial concentration) from a BSA sample by dialysis performed as in Fig 2, except that 150 mM NaCl was included in all the dialysis buffers and the procedure was carried out at r.t. Under these conditions, EDTA remaining in the protein sample was only 33.5 ± 35.0 μM (average value ± SD of 7 determinations), compared to 290.5 ± 23.4 μM EDTA remaining in the absence of NaCl (mean ± SD of 5 determinations). Therefore, these results confirm the previous observations [19] and show the superior effectiveness of EDTA removal by dialysis at physiological ionic strength.

The concentration of EDTA remaining after dialysis will likely not affect the results of studies employing millimolar concentrations of divalent cations [23], which in some cases are even performed in the presence of 1 mM EDTA [9]. Nevertheless, they should be taken into account when performing assays with micromolar concentrations of metals. The presence of EDTA can also affect various protein parameters, like thermal denaturation [20], or induce pH alterations upon addition of divalent cations [24]. Also, in studies assessing the biological effects of dialyzed proteins or cellular or plasma fractions, residual EDTA could be responsible for some of the effects observed.

Potential reasons for the lack of effectiveness of dialysis procedures could include the reported ability of EDTA to form supramolecular aggregates in solution, as well as its capacity to associate with proteins ([25] and references therein). For instance, EDTA has been reported to bind to α-lactalbumin altering the equilibrium between different conformers of the protein and its apparent thermal stability [26].

Size exclusion chromatography using spin desalting columns was among the methods we evaluated to diminish the concentration of EDTA and we found this procedure less effective compared with ultrafiltration. However, we cannot rule out that the use of longer columns with higher resolution may improve the species separation. In addition, care should be exercised with other chromatographic procedures employed in protein purification since EDTA has been reported to be abnormally retained in ion exchange chromatography under certain conditions [24].

## Conclusions

Results presented here show that, despite its low molecular mass (292.24 Da), EDTA is often retained inside dialysis bags with pore-sizes over a hundred times larger, and extending the dialysis time and the number of buffer changes does not seem to allow successful removal of the chelator in those cases. Therefore, determination of the level of EDTA in the dialysates is needed whenever interference of this compound with the intended assays is suspected. For an initial assessment of the potential presence of EDTA, we suggest a fast and cost-efficient method, suitable for small sample amounts, which is based on the competition of EDTA and the zinc-binding compound PAR for complexation of this divalent cation. After testing procedures other than dialysis routinely employed to separate macromolecules from small compounds, we concluded that ultracentrifugation could be a good choice to remove EDTA when necessary. Moreover, when the protein needs to be subjected to dialysis as part of a refolding protocol, the combination of dialysis and ultrafiltration in no particular order renders optimum results in terms of chelator elimination.
